# A CMOS MEMS-based Membrane-Bridge Nanomechanical Sensor for Small Molecule Detection

**DOI:** 10.1038/s41598-020-60057-8

**Published:** 2020-02-19

**Authors:** Yi-Kuang Yen, Chao-Yuan Chiu

**Affiliations:** 1 Department of Mechanical Engineering, National Taipei University of Technology, Taipei, 106 Taiwan; 2 Vanguard International Semiconductor Corporation, Hsinchu, 300 Taiwan

**Keywords:** Drug regulation, Biomedical engineering

## Abstract

Small molecule compounds are necessary to detect with high sensitivity since they may cause a strong effect on the human body even in small concentrations. But existing methods used to evaluate small molecules in blood are inconvenient, costly, time-consuming, and do not allow for portable usage. In response to these shortcomings, we introduce a complementary metal-oxide-semiconductor bio-microelectromechanical system (CMOS BioMEMS) based piezoresistive membrane-bridge (MB) sensor for detecting small molecule (phenytoin) concentrations as the demonstration. Phenytoin is one of anticonvulsant drugs licensed for the management of seizures, which has a narrow therapeutic window hence a level of concentration monitoring was needed. The MB sensor was designed to enhance the structural stability and increase the sensitivity, which its signal response increased 2-fold higher than that of the microcantilever-based sensor. The MB sensor was used to detect phenytoin in different concentrations from 5 to 100  μg/mL. The limit of detection of the sensor was 4.06 ± 0.15  μg/mL and the linear detection range was 5–100  μg/mL, which was within the therapeutic range of phenytoin concentration (10–20  μg/mL). Furthermore, the MB sensor was integrated with an on-chip thermal effect eliminating modus and a reaction tank on a compact chip carrier for disposable utilization. The required amount of sample solution was only 10  μL and the response time of the sensor was about 25  minutes. The nano-mechanical MB sensing method with thermal effect compensation is specific, sensitive, robust, affordable and well reproducible; it is, therefore, an appropriate candidate for detecting small molecules.

## Introduction

Microcantilever (MCL) -based biosensor is one of the label-free molecular sensing methods which has been developed and applied in many fields, such as clinical diagnostics^1,2^, homeland security^3^, environmental monitoring^4,5^ and small molecule detection for therapeutic drug monitoring (TDM) purpose^6–8^. The static-mode operated MCL-based biosensor with electrical readout is especially provided with real-time detection, high sensitivity, and system miniaturization, which is potentially portable for personal diagnosis. In previous studies, the piezoresistive MCL biosensing chips fabricated by using MEMS process was employed for drug concentration monitoring of valproic acid^6^ and phenytoin^7^. Although those works demonstrated enough drug detection ranges for TDM, device sensitivities were limited and additional temperature control systems were needed, which cannot satisfy the demands of personal diagnostic devices.

Many research teams have also developed CMOS-based MCL sensors for detecting biomolecules^9,10^. Huang *et al*. demonstrated a cantilever-based system-on-a-chip (SoC) DNA sensing device realized by a CMOS Bio-MEMS process^9^, but the sensor was limited to operate only in a dry environment to avoid the influence from the ion concentration of the buffer. Besides, Yang *et al*. reported a parallel MCL design in the CMOS-based biosensor for eliminating temperature effect^10^. However, this traditional readout method of dual MCLs with the Wheatstone bridge circuit configurations may compensate for temperature drift to derive accurate measurements, it can bring about fallacious or irreproducible results in a biochemical liquid sample environment^11^. To improve the piezoresistive sensitivity, Yoshikawa *et al*. developed a membrane-type surface stress sensor (MSS) with 2D array consisting of suspended thin film and bridge structures reaching about 100 times higher sensitivity compared to that of the standard piezoresistive MCL^12^. In 2013, Loizeau *et al*. presented the MSS device coated with various polymer layers for detecting the humidity and analyzing the volatile molecules of cancer patients’ breath^13^, but none of the reports showed the MSS devices applied in real liquid sample detection.

In this study, we developed a CMOS MEMS-based membrane-bridge (MB) nanomechanical biosensor for applying to detect small molecule phenytoin as verification of the feasibility and sensitivity of the sensor. Phenytoin is one of the widely used antiepileptic drug in seizure management^14^. Due to its narrow therapeutic index (10–20  µg/mL) and its pervasive daily use, the monitoring and considering potential phenytoin overdose from chronic use is crucial to early management and prevention of further toxicity^15^. The morphology and size of the MB sensing chip was designed and then fabricated by employing the commercial standard manufacturing process operated by Taiwan Semiconductor Manufacturing Company (TSMC) foundry. The MB nanomechanical biosensor with an on-chip real-time thermal effect eliminating method^16^ was operated in static mode. This method not only enhanced the sensitivity of the sensor, but also reduced the overall detection system. The MB nanomechanical biosensor was packaged and performed in a liquid environment by using a designed reaction tank for reducing the sample amount and preventing the interference from the flow field and bubbles of microfluidic device. After that, the MB sensor was demonstrated its capability for phenytoin detection and shows the superior sensitivity than the traditional MEMS based piezoresistive MCL sensor, which may provide a label-free, rapid, sensitive, reliable and miniaturized device for small molecules detection.

## Results and Discussion

### Electromechanical property and thermal effect elimination

The gauge factor represents the ability of the MB sensor to convert mechanical signals into electronic signals. The gauge factor can be calculated by measuring the displacement amount (Δz) of the membrane and the amount of change in the piezoresistance value (Δ*R)* in combination with the known size of the MB structure (See Supporting information S1). From the experimental results, the ratio of Δ*R*/Δz was 2.6793 Ω/μm and the reference piezoresistance *R*_0_ of the MB sensor at 25 °C was 18.275 kΩ. According to the structure and material parameters of MB sensor, the neutral axis position was found to be 1.715 um from the bottom, and the distance from the neutral axis to piezoresistive layer was 1.023 um. Therefore, the gauge factor can be determined to be 21 which was higher than the value of MCL sensor we developed in previous works^17^.

**Figure 1:**
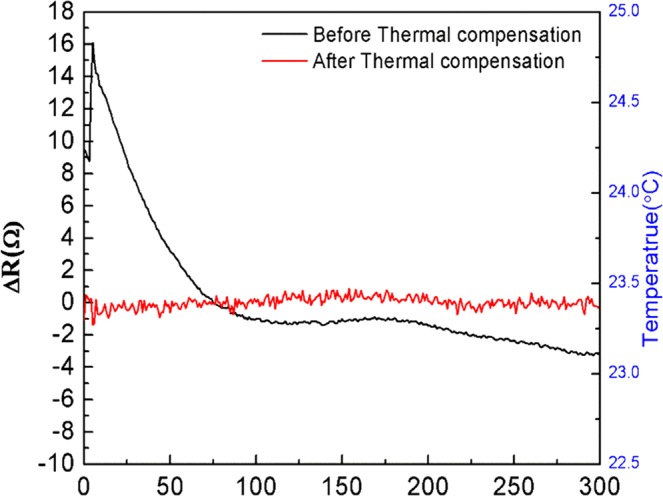
The response signals in the resistance change of the MB nanomechanical biosensor before and after applying the real-time thermal effect eliminating method at room temperature without other temperature control equipment.

Since the MB sensor was composed of multilayer materials, the temperature variation of the environment can greatly affect the detection signals of the sensor. The thermal effects included the temperature coefficient of resistance (TCR) and the material bimorph effect, which caused a major issue of the accuracy for the biomolecular detections of the MB sensor. Therefore, we introduced a thermal effect eliminating method employing an aluminum temperature sensor embedded in the MB sensing chip to measure the temperature surrounding the sensor. Through temperature-gradient experiments operated from 24–28 °C, the relationship of metal and piezoresistive resistance changes correlated with the temperature can be expressed respectively as two quadratic functions (See Supporting information S2). By importing these functions into the self-designed LabVIEW program, the thermal effect compensation can be automatically operated during sample measurements. As for the results, the drifting response signal of the resistance changes due to the fact that the temperature variation can be compensated after applying this method, as shown in Fig. 1. The blank signal of the MB sensor after utilizing the thermal effect elimination was obtained as 164.49 mΩ in the root mean square calculation.

### Surface modification analysis

The X-ray photoelectron spectroscopy (XPS) analysis was carried out for the characterization of the molecular layers immobilized on the gold layer of the MB sensor. Two crucial steps were involved in the surface modification: the linker SAM layer immobilization and the probing antibodies implantation on the linker layer. Therefore, samples of MB sensing chips were prepared respectively, with bare gold layer, with the SAM layer and with immobilized probing antibody. The investigated sample area was 0.6  mm in diameter and the investigated depth was of 2 to 5  nm. By irradiating X-ray on the sample, electrons in specific bound states are excited and then the kinetic energy and number of electrons that escape from the sample surface are measured to obtain the XPS spectra.

**Figure 2:**
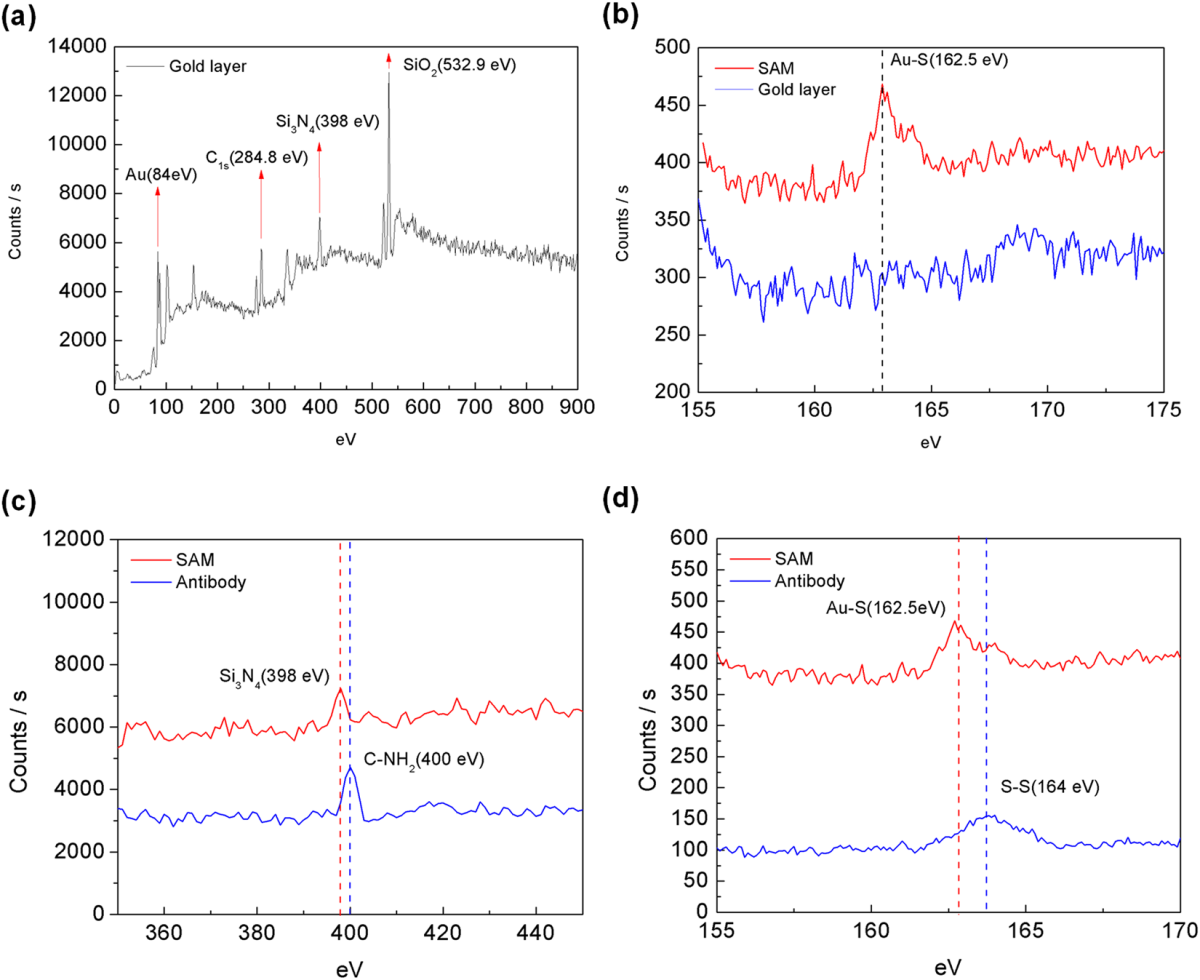
(**a**) The XPS spectrum of an MB sensing chip without any surface molecular modification. (**b**) The sulfur element micro-domain energy spectrum before and after SAM implantation. (**c**) The nitrogen element micro-domain energy spectrum before and after antibody implantation. (**d**) The sulfur element micro-domain energy spectrum before and after antibody implantation.

Figure 2(a) shows the XPS energy spectrum identifying Au (84  eV), Si_3_N_4_ (398  eV) and SiO_2_ (532.9  eV) from the bare gold layer coated MB sensing chip. Figure 2(b) shows the obvious peak at the Au-S bond energy (162.5  eV)^18^ compared with the spectrum of the only gold coated surface, confirming that the SAM layer has been successfully immobilized on the gold surface. Since the antibody consists of a peptide bond and a disulfide bond, the energy peaks of amine group (C-NH_2_) and the disulfide bond (S-S) should be observed in the spectrum. Figure 2(c),(d) show the energy peaks of amino group (400  eV) and the disulfide bond (164  eV)^19^ observed in the spectrum of micro-domain energy amplification compared to the spectrum of the SAM layer coated surface.

### Detection of phenytoin

**Figure 3:**
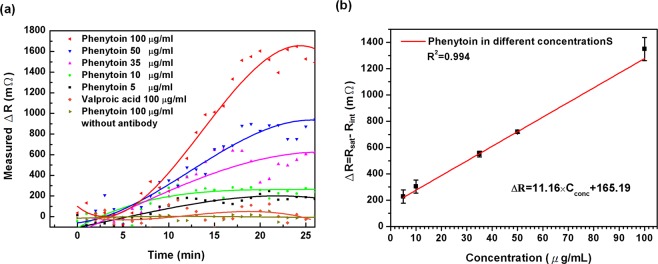
(**a**) Monitoring phenytoin in different concentrations compared to the specificity test with 100  μg/mL other antiepileptic drug valproic acid as well as the control experiment with phenytoin detection without capture antibody. (**b**) Linear regression of the resistance change as a function of phenytoin concentration in DMSO solution. The detection linear region was from 5  μg/mL to 100  μg/mL with a correlation coefficient of 0.994.

In order to verify the *in-vitro* detection capabilities of the system for small molecule phenytoin inspections in the effective therapeutic concentration range (10–20  μg/mL), measurements were made by the anti-phenytoin modified MB sensors against various concentrations of phenytoin. Figure 3(a) presents the change in resistance measured as a function of time when the sensors were exposed to phenytoin concentrations ranging from 5 to 100  μg/mL. Each concentration was measured by using at least three MB sensors. A 10  μL of phenytoin sample was injected into the sensor for each measurement. The signal was recorded after sample injection (0  min); the piezoresistance changes induced by the phenytoin detection began at 5  min, and stabilized at 20  min. The signal data was continuously measured until 25  min after the sample was injected. It was found that the resistance change increased upon exposure to phenytoin, and that the magnitude of the signal was linearly correlated with the concentration of the analytes. In addition, to examining the selectivity of the sensor for phenytoin detection, the sample of the other anti-epileptic drug (valproic acid) in a concentration of 100  μg/mL was measured by an anti-phenytoin modified MB sensor. Moreover, the phenytoin sample of 100  μg/mL was also detected by MB sensor without antibody modification, and the SAM layer was all blocked by the ethanolamine. Figure 3(a) shows weak or no response of devices that measured the non-specific molecules (valproic acid) or carried no antibodies.

Figure 3(b) shows an averaged calibration curve of response signals for phenytoin detection in different concentrations between 5 and 100  μg/mL. The value of response signal in resistance change (ΔR) related to tested phenytoin concentration was obtained by subtracting average value of piezoresistance (R_sat_) in the last 5  min from the average value of piezoresistance (R_int_) in the first 5  min. A linear relationship can be found between the resistance change ΔR and phenytoin concentrations C_conc_, which is expressed as ΔR = 11.16 × C_conc_ + 160.25. This indicates the sensor sensitivity being 11.16 ± 0.42 mΩ per μg/mL with a regression coefficient of 0.994. In this sensing system, the magnitude of the root-mean-square of the blank signal after thermal effect elimination has been measured was 114.62 mΩ and the standard deviation of the blank signal was 30.32 mΩ. Therefore, the limit of detection (LOD) of the MB biosensor for phenytoin detection is calculated as the blank signal plus 3 times of standard deviation of the blank to be 205.58 mΩ and is expressed in the concentration of 4.06  μg/mL from the calibration curve. The linear detection range and the LOD of the MB biosensor have been proven to be capable of detecting phenytoin in the therapeutic concentration range (10–20  μg/mL). The limit of quantification (LOQ) is commonly defined as the blank signal plus 10 times the standard deviation of blank and calculated to be 417.82 mΩ and is expressed in the concentration of 23.08  μg/mL.

### Comparison of signal responses of MB and MCL biosensor

**Figure 4:**
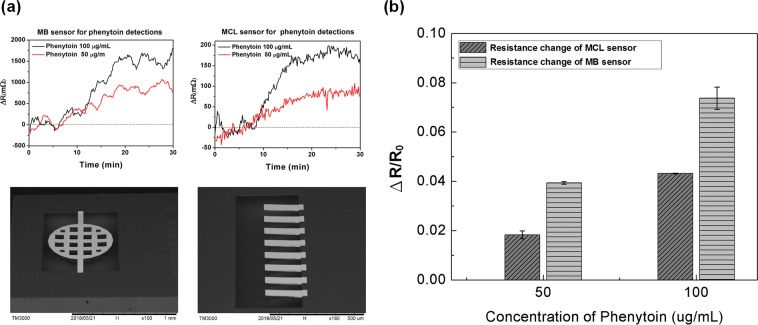
(**a**) Resistance changes in signal of phenytoin monitoring in the concentration of 50 and 100  μg/mL by MB and MCL sensors respectively. (**b**) Comparisons of the signal responses in the resistance change ratio between MCL and MB sensors for 50 and 100  μg/mL phenytoin detection. The response of the MB sensor increased about 2-fold compared to that of the MCL sensor.

In this study, the MB biosensor’s sensitivity was improved not only by expanding the sensing area, but also by increasing the signal response by superimposing the piezoresistance change at both ends of the MB structure. Two different concentrations of phenytoin at 50  μg/mL and 100  μg/mL were respectively measured by both using CMOS MEMS-based MB and MCL biosensors. The MCL used in the experiment has a piezoresistance of one-third of the total length and a line width of 4 μm. The dimensions of the MCL were 200 μm long, 43 μm wide and about 3.8 μm thick, as shown in the bottom right of Fig. 4(a). As well, the total length of the MB structure was 1000 μm, the diameter of circular membrane was 600 μm and the stiffness of MB was six times higher than that of the MCL. In the upper part of Fig. 4(a), signal changes of MB and MCL biosensors for measuring phenytoin at concentrations of 50  μg/mL and 100  μg/mL are shown respectively. The values of resistance change of MB biosensors were respectively 719 mΩ and 1396 mΩ on average, while those of MCL biosensors were respectively 76 mΩ and 179 mΩ on average. Figure 4(b) shows the resistance change ratio of MCL phenytoin sensor compared with that of MB sensor respectively at the concentration 50 and 100  μg/mL. The signal response ΔR/R_0_ was the resistance change ratio of MCL and MB sensors with respect to phenytoin concentrations. Since the initial resistance R_0_ varied between MCL and MB sensors, in order to compare the real improvement of signal responses, the resistance changes of both sensors are divided by their own initial resistances. It can be found that the response sensitivity of the MB sensor for phenytoin detection increased about 2-fold compared to that of the MCL sensor.

### Comparison with other methods

The methods of small molecule phenytoin detection are listed in Table 1. Most chromatography and fluorescence label detection methods have high sensitivity, high accuracy and broad specificity ability to detect complex mixtures. However, these methods need costly equipment, specialists for operation, sample preprocessing and prolonged measurement periods. The merits of the surface plasmon resonance (SPR) imaging-based phenytoin sensor are label-free, sensitive, rapid response, and portable, but its drawbacks are requirements of sample precondition and specialist operation as well as expensive inspection costs. Although the MCL sensor shows its capability of label-free detecting phenytoin with the advantages of rapid response, portability and low cost, its sensitivity is not high compared to other methods. The MB sensor not only has the merits of the MCL sensor, but also increases the sensitivity by expanding the reaction area. Moreover, the bridge structure makes the sensor more stable due to the increased stiffness. Therefore, the MB sensor demonstrates capable potential as a point-of-care device for TDM application.

**Table 1:** The comparison of reported methods for phenytoin detection.

Method	Sample	Linear range (µg/mL)	LOD (µg/mL)	Reference
^1^CMOS MEMS based MB biosensor	DMSO	5–100	4.06	This work
^2^MEMS based MCL biosensor	DI water/ serum	10–80	9.5	^7^
^3^SPR imaging	Human saliva	-	50 nM	^21^
^4^GC-TSD	Plasma	0.05–40	0.05–0.2	^22^
^5^HPLC	Serum /plasma	2.2–48.5	2.2	^23^
^6^FPIA	Serum	0–40	0.5	^24,25^

^1^MB, membrane-bridge, ^2^MCL, microcantilever; ^3^SPR, surface plasmon resonance; ^4^GC-TSD, gas chromatography with thermionic specific detection; ^5^HPLC, high-performance liquid chromatography; ^6^FPIA, fluorescence polarization immunoassays.

## Conclusion

In this work, we demonstrate nanomechanical MB sensing device fabricated by using the standard CMOS MEMS process for a selective detection and quantitative analysis of small drug molecule phenytoin. The method achieves appropriate responses of phenytoin concentrations in the effective therapeutic range in the aqueous samples. We integrated the Eppendorf on the PCB chip carrier and pipetted a 10  μL analyte solution into the sensing chip, avoiding the bubble obstruction from the liquid handling microchannel. We applied the on-chip thermal effect elimination to decrease temperature drift signals to enhance the accuracy and reproducibility of the sensor. The surface analysis results indicated that the linker molecule and the probing antibody were immobilized on the surface of the sensor. The control examinations were performed to confirm the specificity of the sensor in phenytoin detection. With respect to the performance of device, we found the detection response of the MB sensor was 2-fold higher than that of the MCL sensor with the same phenytoin concentrations. The sensitivity of the MB sensor was verified to have capability for sensing of phenytoin in liquid sample, but still can be improved by considering the length and shape design of the piezoresistor in the bridge end structure. This work was compared with the modern methods for analysis of phenytoin and showed its high feasibility in clinical application; however, this method needs further examinations with real human serum and blood samples.

## Materials and Methods

### Device fabrication

**Figure 5:**
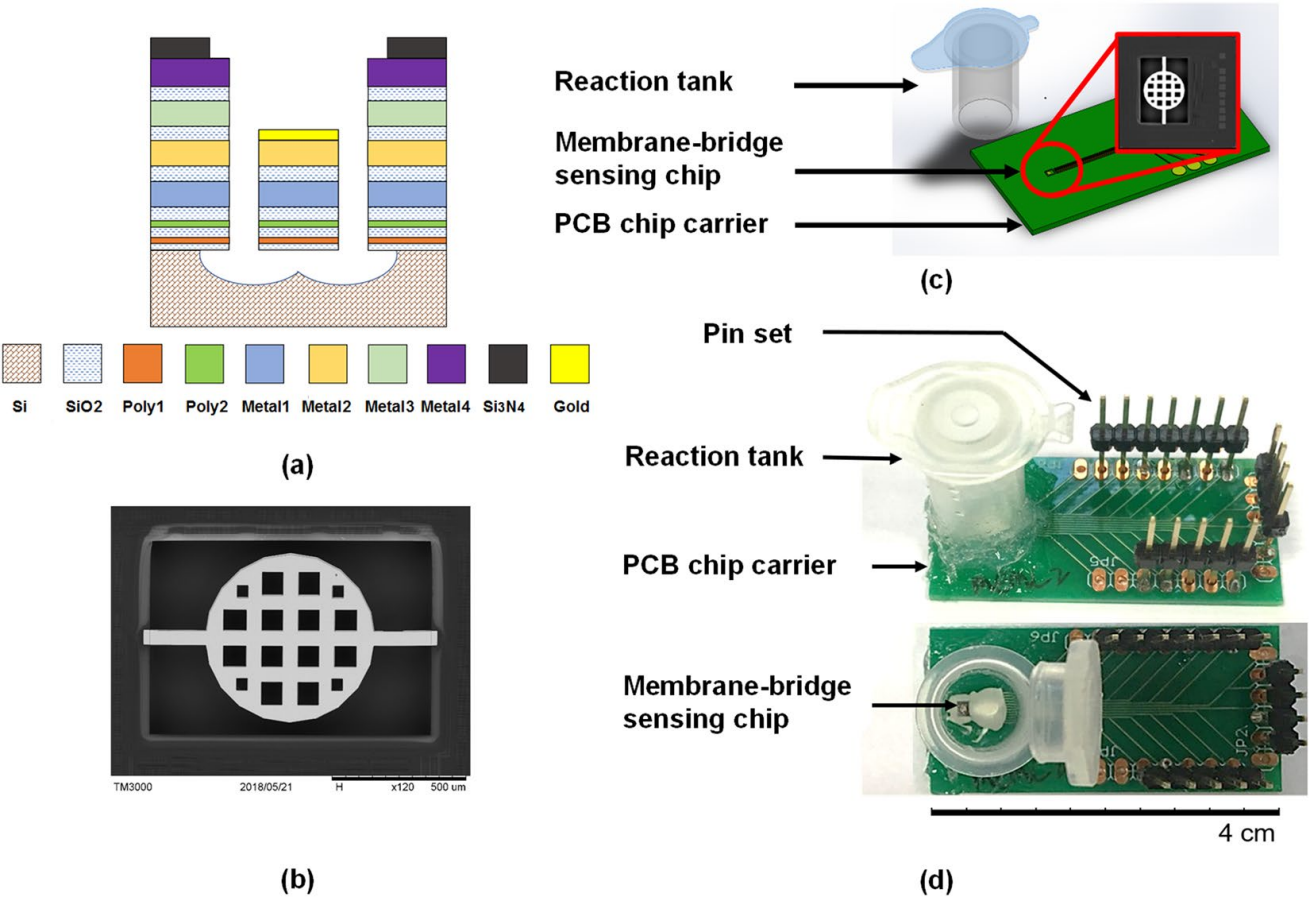
(**a**) The schematic representation of the standard TSMC 2P2M CMOS process for fabricating piezoresistive MB sensing chip dies. (**b**) The SEM image top view of the CMOS MEMS- based MB sensing chip. The diameter of the circular membrane structure was 600 μm and square holes in the membrane were designed for the structure releasing process. Piezoresistors were embedded in the two ends of the MB structure. (**c**) The schematic diagram of the specific module, including reaction tank and electrical readout for phenytoin detection with microscale volume of sample. (**d**) The top and side views of the entity picture of MB disposable device for phenytoin detection.

The membrane-bridge sensing chip was fabricated by using the TSMC COMS MEMS 0.35UM 2P4M standard process with the size of 1.5 mm^2^. In the 2P4M standard process, multilayer materials comprised two polysilicon layers and four aluminum layers, as shown in Fig. 5(a). The structure of the MB sensor was 2P2M in order to increase the sensitivity. The Poly1 layer was designed as the lower protective layer of the bridge membrane, and the Poly 2 layer as the piezoresistive layer; Metal 1 and Metal 2 layers were the structural layers. Through the post-process, a gold film was deposited on top of Metal 2 layer, which was highly biocompatible and suitable for modifying the self-assembled monolayer or bio-recognition molecules so that the MB sensor was functionalized to achieve detection. The dimensions of the fixed end of the MB sensor were 3.8 μm thick, 43 μm wide and 200 μm long, while the circular membrane was 300 μm in radius. In order to ensure the complete release of the MB sensing chip in the post-process, the ratio of etching holes and the spaces in the membrane structure was designed as 2:1. For the purpose of eliminating the thermal effect of the sensor, the Metal 1 aluminum wire was used as the temperature sensing component. The metal wire has better resistance to temperature stability and higher linearity than the piezoresistive material^20^. The size of the metal wire was 1 μm in width and 2100 μm in length, and the resistance value of the metal wire at 25 °C was calculated as 147.7 Ω. The SEM image of the MB chip configuration is shown in Fig. 5.

### Detection system integration

For small molecule concentration measurements, experiments must be conducted in liquids such as acid, alkali, organic, etc. Therefore, the detection system should be packaged as chemical resistant to avoid leakage and short circuit during the measurement. The printed circuit board (PCB) chip carrier was designed by using the Eagle PCB software. The size of PCB was 20  mm wide, 40  mm long and 0.6  mm thickness. The MB sensing chip was first adhered to the PCB with DOW CORNING ® SE4486 adhesive which not only has the function of insulation and waterproofing, but also has good thermal conductivity. Next, the chip was wire bonded onto the contacts of PCB and the exposed contacts were insulated from the liquid by using DOW CORNING ® SE4486 silicone glue.

The measurement using microfluidics to flow samples to the sensing area needs an extra pumping system. Moreover, bubbles could be induced in the microchannel during pumping flow, thereby possibly causing an unrecoverable deflection of the MB and leading to the unrecognized signals. Thus, the reaction tank was introduced and manufactured by using a 1.5  mL Eppendorf which can withstand high temperature and high-pressure sterilization and has chemical resistance. The Eppendorf was cut into a tapered front end by a cutter to form a capped column having an inner diameter of 10  mm and a volume of 1.2  mL. Next, the reaction tank was adhered to the PCB with DOW CORNING ® 3140 waterproof glue, so that the tank completely covered the sensing chip to form a closed reaction chamber. The schematic plot and entity picture of the whole detection package of CMOS MEMS-based MB nanomechanical sensor are shown in Figs. 5(c) and 1(d).

### Surface functionalization and sample preparation

**Figure 6:**
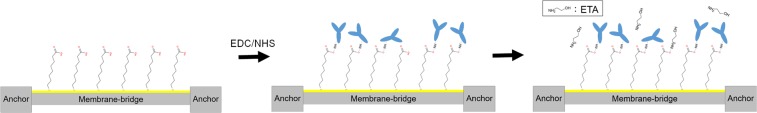
The schematic diagram of procedures for surface functionalizing of MB sensor. Step 1: 8-mercaptooctanoic acid SAM was modified on the gold surface. Step 2: the SAM layer was activated by EDC/NHS followed by the immobilization of anti-phenytoin probing molecules. Step 3: ethanolamine was used to block the unbonded SAM molecules.

To functionalize the gold surface of the sensing chip (as shown in Fig. 6), the first step was to employ the self-assembly monolayer (SAM) (SH(CH_2_)_7_COOH) as a linking layer between the probing molecules and the MB sensor surface. Most of the chemicals and solutions were purchased from Sigma-Aldrich, Inc. The 8-mercaptooctanoic acid (Sigma-Aldrich 675075) was formulated with 99.8% ethanol (Sigma-Aldrich 32221) to a concentration of 100  mM. The solution was then injected onto the sensor surface and incubated in the reaction tank for 24  h to ensure formation of the SAM. After the incubation, deionized water was applied to rinse three times to remove unbonded molecules. The second step was to activate the carbonyl functional group of the SAM layer. A 400  mM EDC (N-(3-Dimethylaminopropy1)-N’-ethylcarbodiimide hydrochloride) was mixed with a 100  mM NHS (N-Hydroxysuccinimide) (Sigma-Aldrich 130672) in a volume ratio of 1:1. The mixed solution was driven to the chip surface and incubated for 40  min, followed by injecting the PBS (Phosphate buffered saline) buffer solution onto the chip surface for 10  min and rinsed three times. Thirdly, the 100  μg/mL of the capture molecule (sheep polyclonal phenytoin antibody, Fitzgerald Industries International 20–1592) solution was applied to an activated chip surface and incubated for 3  h to achieve antibody immobilization. PBS solution was again injected to the surface for 10  min and rinsed three times. The final step was blocking the SAM which had not bonded to the capture antibody by applying 1 M ethanolamine (Sigma-Aldrich 150514–1) for increasing the accuracy of the measurements. This was followed by rinsing three times with pure ethanol, so the MB sensor was ready for phenytoin detection. Regarding the detection sample preparation, the anti-epileptic drug phenytoin was diluted to a known concentration with DMSO (Dimethyl Sulfoide) (Merck Millipore 317275). A 10  μL of phenytoin (Abcam ab143201) sample solution was then pipetted into the MB sensor for the measurement, while the lid of the reaction tank should be capped to prevent evaporation.

### Experimental Instruments

An X-ray photoelectron spectroscopy (XPS, VG Scientific ESCALAB 250) was employed to confirm the adsorption of SAM and probing molecules on the surface of the MB sensing chip. A temperature control platform (Unice E-O Services Inc. 10TEC-150) was used for the calibration of the temperature sensor embedded in the sensing chip for self-thermal effect elimination. A multi-function digital meter (NI PXI-4071) with a multiplexer switch module (NI PXI-2503) was utilized for signal capturing and recording. Device dimension confirmation was scanned by using a scanning electron microscope (SEM, JeoL JSM-7800F Prime).

## Supplementary information

Supplementary information.
