# Supplementary material for: A CMOS MEMS-based Membrane-Bridge Nanomechanical Sensor for Small Molecule Detection
Source: Sci Rep. 2020 Feb 19;10:2931. doi: 10.1038/s41598-020-60057-8 (PMC7031247; doi:10.1038/s41598-020-60057-8)
Supplement: Supplementary file 1 — Supplementary information. [file 41598_2020_60057_MOESM1_ESM.docx]

Supporting Information

A CMOS MEMS-based Membrane-Bridge Nanomechanical Sensor for Small Molecule Detection

Yi-Kuang Yen ^1^, * and Chao-Yuan Chiu ^2^

^1^ Department of Mechanical Engineering, National Taipei University of Technology, Taipei 106, Taiwan

^2^ Vanguard International Semiconductor Corporation, Hsinchu 300, Taiwan

^*^Corresponding author: Yi-Kuang Yen (ykyen@ntut.edu.tw)

**S1. The determination of the gauge factor of MB sensor**

The gauge factor is defined as the ratio of the resistance change to the axial strain. Since the aspect ratio of the piezoresistive layer design is very large, the lateral change can be omitted. The gauge factor can be expressed as:

$G=\frac{\Delta R/\mathrm{R}_{0}}{\varepsilon}$ (S-1)

where $\Delta$R represents the amount of change in resistance value, R_0_ represents the value of the reference resistance, and ε represents the axial strain. Assume that the total length of the bridge film is 2L, the piezoresistive length is λ, the distance between the piezoresistive layer and the neutral axis of the membrane-bridge (MB) structure is Z_R_, and the moment of the MB subjected to the end point force is M(x). When the area moment of inertia of the MB is I, the stress distribution when the MB structure subjected to force is:

$\sigma(x)=\frac{M(x)Z_{R}}{I}$ (S-2)

Calculate the average stress of piezoresistance using the above equation:

$\sigma=\frac{1}{\lambda}\int_{0}^{\lambda} \sigma(x)dx$ (S-3)

$\sigma=\frac{1}{\lambda}\int_{0}^{\lambda} \frac{M(x)\mathrm{Z}_{R}}{I}dx$ (S-4)

$\sigma=\frac{FZ_{R}}{4I\lambda}\int_{0}^{\lambda} [3L-2x])dx=\frac{FZ_{R}}{4I}(3L-\lambda)$ (S-5)

Next, calculate the strain ε of the bridge film by Hooke's law from (S-5):

$\varepsilon=\frac{FZ_{R}}{4EI}(3L-\lambda)=\frac{Z_{R}}{4EI}(3L-\lambda)k\Delta z$ (S-6)

Where F is the concentration force applied to the center of MB, E is the Young's modulus, and k is the spring constant. The relationship between the stiffness coefficients of the MB structure is:

$k=\frac{192EI}{8L^{3}}$ (S-7)

Substituting the equation (S-7) into the equation (S-6) can be obtained:

$\varepsilon=\frac{6Z_{R}}{L^{3}}(3L-\lambda)\Delta z$ (S-8)

Finally, the equation (S-8) is substituted (S-1) to obtain the gauge factor:

$G=\frac{\Delta R}{R}\times\frac{L^{3}}{6Z_{R}(3L-x)\Delta z}$ (S-9)

According to the equation (S-9), the gauge factor of the MB sensor can be calculated by measuring the displacement change ($\Delta$z) of the center of the MB structure and the amount of change in the resistance value ($\Delta$R) in combination with the known size of the MB. The schematic diagram of the experiment is shown as Figure S1, control the amount of displacement ($\Delta$z) of the probe at the center of the membrane, and simultaneously measure the amount of resistance change ($\Delta$R) to obtain their ratio ($\frac{\Delta R}{\Delta z}$).


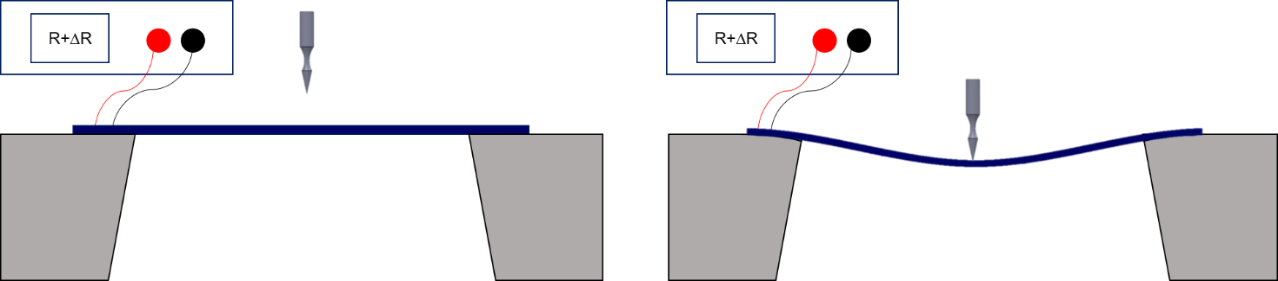


**Figure S1.** Schematic diagram of the gauge factor measurement experiment.

The experimental results of the displacement ($\Delta$z) and resistance change ($\Delta$R) of the MB sensor are as follows:





**Figure S2.** The plot of the measuring result of displacement ($\Delta$z) is related to the resistance change ($\Delta$R) of MB structure.

It can be obtained from the experimental results that $\frac{\Delta R}{\Delta z}=2.6793(\Omega/\mu m)$and the reference resistance R_0_ of the MB at 25 ° C is 18.3 (kΩ).

The distance Z_R_ of the piezoresistive layer to the neutral axis of the MB structure can be calculated by firstly obtaining the neutral axis position Z_N_ from the material parameters of the MB. The formula is described as follows:

$Z_{N.\text{A}}=\frac{\sum_{i} E_{i}z_{i}h_{i}}{\sum_{i} E_{i}h_{i}}$ (S-10)

where E_i_ is Young's modulus of the i-th layer material and z_i_ is the neutral axis position of the i-th layer material. h_i_ is the thickness of the i-th layer. The material parameters of the MB structure are obtained from TSMC. Therefore, we can obtain the neutral axis position of the MB is 1.715 μm from the bottom, that is, Z_N.A_=1.715 μm by substituting material parameters in equation (S-10). Then subtract the position of the neutral axis from the position of the piezoresistive layer to obtain the distance from the piezoresistive layer to the neutral axis which is Z_R_=1.023 μm. Substitute the above parameters into equation (S-9) can obtain the gauge factor G=20.5.

**S2 Thermal effect self-compensation system** ^1^

In order to establish a thermal effect self-compensation system, firstly, functions of the temperature corresponding to the resistance value of the on-chip temperature sensor and the MB sensor must be established.

The MB sensing chip adheres to the temperature control platform (Honghui Photoelectric 10TEC-150) with a thermal paste. Temperature gradient measurements are determined by using a multi-function digital meter DMM (NI PXI-4071) with a division of labor and matrix relay (NI PXI-2503). The setting of temperature control is usually from 19 to 28 °C and is spaced at 1.00 °C. For each measurement, the temperature is controlled to be last for 15 minutes. This temperature range is slightly adjusted depending on the ambient temperature during the experiment. When the temperature control platform starts to operate, simultaneously the multi-function digital meter records the resistance signals of the on-chip temperature sensor and the MB sensor at intervals of one data point every 10 seconds. When the temperature-to-resistance change was obtained, temperature functions of the on-chip temperature sensor and the MB sensor can be established as following equations (Figure S3).

$R_{Metal}=a(\mathrm{T}-\mathrm{T}_{0})^{2}+b(\mathrm{T}-\mathrm{T}_{0})+c$ (S-11)

$R_{membrane-bridge}=d(\mathrm{T}-\mathrm{T}_{0})^{2}+e(\mathrm{T}-\mathrm{T}_{0})+f$ (S-12)

After obtaining the temperature functions, the instantaneous temperature of the MB sensing chip can be got by substituting the resistance value of the on-chip temperature sensor into equation (S-11) during the experiment. By taking instantaneous temperature into the equation (S-12) and calculating the resistance value of the MB sensor affected by the current temperature. The thermal effect of the ambient temperature on the MB sensor can be corrected to obtain real bio/chemical signals in the measurement. The thermal self-compensation equation is as follows:

$R_{real}=R_{measure}-R_{Thermal}$ (S-12)

where *R_measure_* is the resistance value measured by the digital meter, and *R_real_* is the real signal after the MB sensor is thermally compensated.


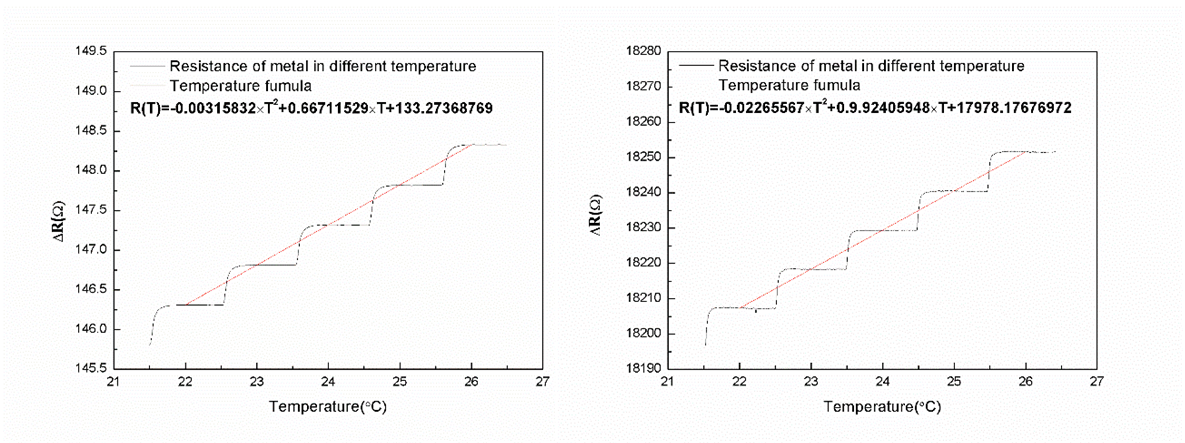


**Figure S3.** Plots of the resistance change versus temperature of the on-chip temperature sensor and MB sensor.

**Reference:**

1. Ku, Y.-F., Huang, L.-S. &Yen, Y.-K. A real-time thermal self-elimination method for static mode operated freestanding piezoresistive microcantilever-based biosensors. *Biosensors* **8**, (2018).
